# Risk factors of chronic kidney disease among type 2 diabetic patients with longer duration of diabetes

**DOI:** 10.3389/fendo.2022.1079725

**Published:** 2022-12-09

**Authors:** Khalid Siddiqui, Teena P. George, Salini S. Joy, Assim A. Alfadda

**Affiliations:** ^1^ Strategic Center for Diabetes Research, College of Medicine, King Saud University, Riyadh, Saudi Arabia; ^2^ Department of Medicine, College of Medicine, and King Saud University Medical City, King Saud University, Riyadh, Saudi Arabia; ^3^ Obesity Research Center, College of Medicine, King Saud University, Riyadh, Saudi Arabia

**Keywords:** diabetic kidney disease (DKD), diabetic nephropathy, longer duration of diabetes, risk factors, high prevalence of diabetes

## Abstract

**Background:**

Chronic kidney disease (CKD) in patients with type 2 diabetes mellitus (T2DM) is the major cause of end stage renal disease, characterized by proteinuria with a subsequent decline in glomerular filtration rate. Although hyperglycemia is the major risk factor for the development and progression of kidney disease among diabetic patients, many other risk factors also contribute to structural and functional changes in the kidneys. As recommended by Kidney Disease Improving Global Outcomes (KDIGO), CKD classification based on cause and severity, links to risk of adverse outcomes including mortality and kidney outcomes.

**Objective:**

The aim of this study is to investigate the involvement of risk factors associated with the severity of CKD among participants with longer duration of diabetes. This study also aims to find whether number of risk factors vary among risk of CKD progression categories based on KDIGO classification.

**Material and methods:**

This cross-sectional study retrospectively selected 424 participants from type 2 diabetic cohort and categorized them based on the classifications for the diagnosis of kidney diseases in patients with diabetes, according to the KDIGO guidelines. Odds ratios and 95% CI of each risk factors according to severity of renal disease were determined.

**Results:**

Based on KDIGO classification, participants with type 2 diabetes (T2D) were categorized in to low risk (n=174); moderately increased risk (n=98); and high/very high risk (n=152). Type 2 diabetic participants with risk factors such as, hyperlipidemia, hypertension, DM duration ≥15 years and diabetic retinopathy showed a high/very high risk of CKD progression when compared with low-risk category. While T2D participants with risk factors such as, lack of exercise, hypertension, and diabetic retinopathy showed a moderately increased risk of CKD progression. In addition, participants with highest number of risk factors were significantly distributed among high/very high risk of CKD progression category.

**Conclusion:**

This study findings conclude that patients with T2DM and duration of ≥15 years, hyperlipidemia, hypertension and diabetic retinopathy have an increased prevalence of advanced CKD. In addition to this, increased number of risk factors could be an indicator of the severity of CKD in T2D.

## Introduction

Diabetes mellitus (DM) is a chronic metabolic disorder characterized by elevated blood glucose level and associated with number of complications including acute metabolic and long-term vascular complications. Type 2 diabetes mellitus (T2DM) is the most common type of diabetes worldwide (1). Chronic kidney disease (CKD) in patients with T2DM is the major cause of end stage renal disease, characterized by proteinuria with a subsequent decline in glomerular filtration rate (2). The great increase in the prevalence of diabetes, significant morbidity and mortality associated with kidney disease among patients with diabetes and burden of healthcare cost has led to research imperative in near future (1).

The development and progression of diabetic kidney disease (DKD) involves the complex interplay of many factors contributing to the structural and functional changes in the kidneys. Chronic hyperglycemia is a crucial factor responsible for the development of kidney disease in patients with DM and lead to progressive glomerular and tubular damage, negatively impacting on health outcomes. CKD in patients with DM is defined as elevated urinary albumin excretion or impaired renal function or both and approximately half of the patients with T2D develop renal diseases (3). KDIGO guidelines recommend that the combined assessment of eGFR and albuminuria status provide more accurate evaluation as being at low, moderately increased, high or very high risk of worsening kidney function (4).

Besides hyperglycemic condition, the combination of other factors including hypertension, dyslipidemia, genetic predisposition, obesity, lifestyle factors are also contributing to the development and progression of kidney disease among patients with diabetes. The prevention and management of CKD in patients with DM might depend on lifestyle modifications and pharmacological strategies such as combined targeted therapies for hyperglycemia, hypertension, albuminuria, hyperlipidemia, and regular use of reno protective agents, which might provide synergistic effect in controlling and minimizing the effect of these factors (5). Recently a joint group of American Diabetes Association (ADA) and KDIGO provide evidence-based recommendations and guidelines to improve clinical outcomes of people with diabetes and CKD which includes CKD screening and diagnosis, glycemia monitoring, lifestyle therapies, treatment goals and pharmacologic management (6). The impact of non-modifiable factors such as age, gender and duration of diabetes are also critical (3, 7).

We hypothesized that in high-risk population, the identification and efficient management of the modifiable risk factors may prevent or delay the development of CKD in patients with diabetes. In this population, there is a general lack of consensus regarding the non-modifiable and modifiable risk factors and comorbidities that are associated with risk of CKD in diabetes. This study aims to investigate the involvement of modifiable and non-modifiable risk factors and other comorbid conditions linked with severity of CKD in T2D cohort with longer duration of diabetes. This study also aims to examine whether number of risk factors vary among risk categories of CKD.

## Materials and methods

### Study population

The data of the study population were collected retrospectively from a previous cohort study conducted at University Diabetes Center, King Saud University Medical City (KSUMC), Riyadh, Saudi Arabia during the year 2014-15 (8). The study was approved by Institutional Review Board (IRB) at College of Medicine, King Saud University (IRB/E-19/3969) and was carried out in accordance with the declaration of Helsinki (8).

The inclusion criteria of this study were as follows: (1) Saudi nationals with T2DM (2) age between 35 and 70 years (3) greater than 10 years of diabetes duration. Exclusion criteria were as follows: (1) pregnant women (2) other causes of renal impairment which includes glomerulonephritis, interstitial nephropathy, vasculitis, malignant hypertension, pelvicalyceal infection, bilateral cortical necrosis, amyloidosis; (3) patients with abnormal liver function (4) patients who take the medications that might affect kidney functions; and (5) end stage renal disease (ESRD) patients (6) patients with cancer.

**Figure 1:**
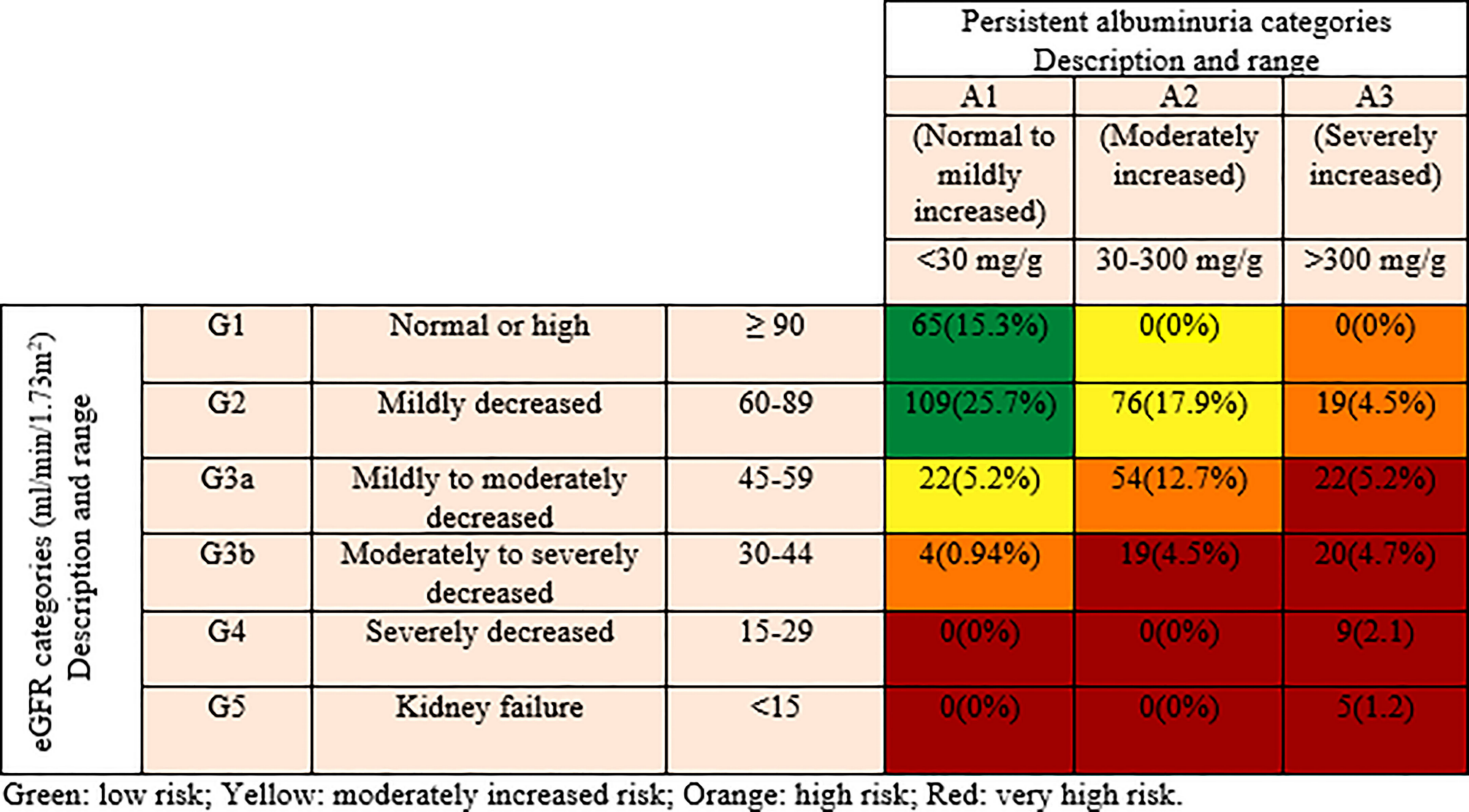
Distribution of participants according to the Kidney Disease Improving Global Outcomes (KDIGO) classification.

Retrospectively selected 424 participants and categorized based on the classifications for the diagnosis of kidney diseases in patients with diabetes, according to KDIGO guidelines. The selected participants were subdivided into three groups according to the severity of risk, namely; low, moderately increased and high/very high risk. Six eGFR categories were included namely; G1, G2, G3a, G3b, G4 and G5 (G1 represents ≥90, G2 represents 60–89, G3a represents 45–59, G3b represents 30–44, G4 represents 15–29 and G5 represents <15 ml/min/1.73 m^2^ of eGFR). Similarly, based on the values of ACR, three categories were included namely; A1, A2 and A3 (A1 represents <30 mg/g, A2 represents 30-300 mg/g and A3 represents >300 mg/g of ACR). Low-risk category comprises of G1A1 and G2A1; moderately increased risk category includes G1A2, G2A2 and G3aA1; and high/very high risk category comprises of G1A3, G2A3, G3aA2, G3bA1, G3aA3, G3bA2, G3bA3, G4A3, and G5A3 (9, 10) (**Figure 1** **)**. 

Risk factors of DKD were determined according to National Kidney Foundation guidelines and Rubeaan et al. (11, 12). Data of non-modifiable risk factors (age, gender, diabetes mellitus duration (DM duration)) and modifiable risk factors (glycemic control (fasting blood sugar (FBS), HbA1c), blood pressure (systolic blood pressure (SBP) and diastolic blood pressure (DBP)), lipids (total cholesterol, triglyceride, HDL cholesterol, LDL cholesterol) obesity (body mass index (BMI), smoking, lack of physical activity) were collected from previous study records (8).

Diagnosis of T2D was based on the ADA criteria or reported to be taking treatment for diabetes (13). The diabetic neuropathy was evaluated by assessing upper and lower extremities of nerve conduction velocity. The presence of at least one definite microaneurysm in any field photographed was considered as the criterion for the diagnosis of diabetic retinopathy (14). Hypertension is diagnosed when blood pressure is consistently ≥130 and/or ≥80mm Hg or being managed with antihypertensive medications such as angiotensin II receptor antagonist, thiazide diuretic, angiotensin-converting enzyme inhibitor, beta blocker, and calcium channel blockers (15). CKD-EPI creatinine equation was used to calculate eGFR (16). 

Statistical analysis was performed using statistical software, SPSS version 21.0 (IBM Corp., Armonk, NY, USA). The categorical data were summarized as percentage and the continuous data were presented as mean and standard deviation and the difference were analyzed using one-way ANOVA (analysis of variance). The categorical variables were analyzed by using Chi-square test. Odds ratio (OR) were used for association of risk factors with severity of kidney disease. OR was calculated using online statistical calculator (https://www.medcalc.org/calc/odds_ratio.php) and expressed in 95% confidence intervals and graphically represented in the form of forest plot. Low risk group was used as reference group to calculate the OR. The p-value was taken as significant at p<0.05.

## Results

The selected 424 T2DM participants were subdivided in to three groups according to the severity of kidney diseases (low risk, n=174; moderately increased risk, n=98; high/very high risk, n= 152). The three groups were similar in BMI, DBP, HDL and LDL cholesterol whereas age, DM duration, SBP, glycemic parameters (FBS, HbA1c), and lipid parameters (total cholesterol and triglycerides) were significantly differed among three groups (**Table 1**).

**Table 1:** Demographic and clinical characteristics of patients with type 2 diabetes according to KDIGO classification.

Parameters	Low risk (n=174)	Moderately increased risk (n=98)	High/very high risk (n=152)	p value
Age (years)	54 ± 6.0	55.9 ± 6.0	55.9 ± 6.4	0.01
Gender (male, n %)	74 (42.5)	47 (48)	68 (44.7)	0.68
DM duration (years)	16.8 ± 4.5	18.9 ± 6.0	19.5 ± 5.9	<0.001
Hypertension, n (%)	95 (54.6)	75 (76.5)	128 (84.2)	<0.000
Hyperlipidemia, n (%)	139 (79.9)	84 (86.6)	141 (92.8)	0.004
BMI (kg/m^2^)	32.7 ± 5.7	31.7 ± 5.1	32.3 ± 5.9	0.38
SBP (mm Hg)	130.4 ± 16.9	138.6 ± 18.0	143.6 ± 20.9	<0.001
DBP (mm Hg)	72.6 ± 10.0	75.2 ± 10.6	74.3 ± 11.7	0.12
FBS (mg/dl)	187.8 ± 74.2	228.5 ± 94.8	243.1 ± 103	<0.001
HbA1c (%)	10.0 ± 1.3	10.5 ± 1.5	10.9 ± 2.1	<0.001
Total cholesterol (mg/dl)	172.5 ± 37	182.7 ± 44.0	198.6 ± 54.2	<0.001
Triglyceride (mg/dl)	151.2 ± 61.0	186.5 ± 89.7	208.7 ± 94	<0.001
HDL cholesterol (mg/dl)	44.8 ± 10.5	46.7 ± 12.1	47.4 ± 13.5	0.13
LDL cholesterol (mg/dl)	130.8 ± 40.6	134 ± 40.5	138.2 ± 50.9	0.32

Data represents in mean ± standard deviation and percentage. DM duration (diabetes mellitus duration), BMI (body mass index), SBP(systolic blood pressure), DBP(diastolic blood pressure), FBS (fasting blood sugar), HDL cholesterol (high density lipoprotein cholesterol), LDL cholesterol (low density lipoprotein cholesterol), P value <0.05 is statistically significant.

**Figure 2:**
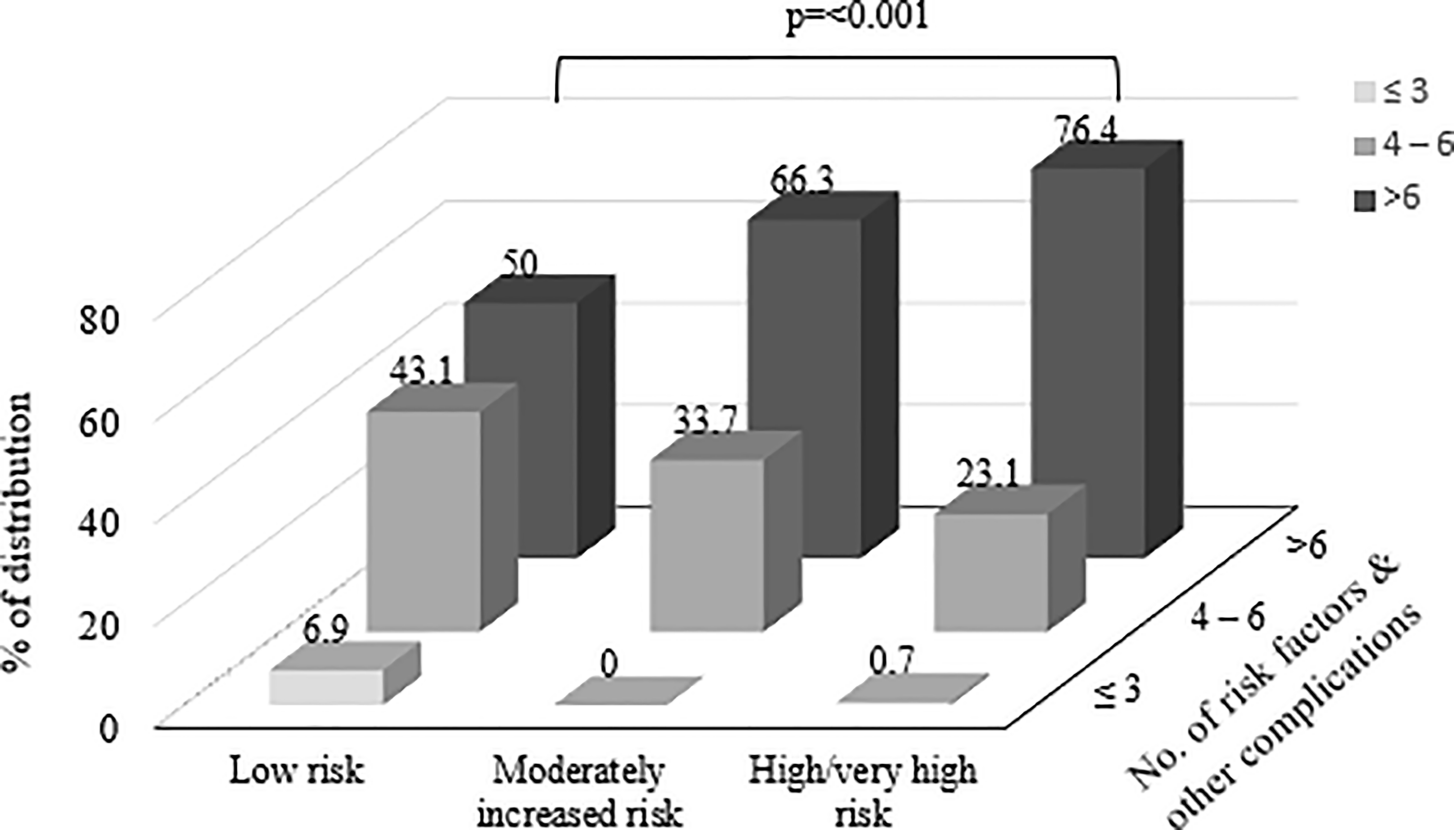
Shows the percentage of distribution of participants according to the number of risk factors and other complications among different risk categories based on Kidney Disease Improving Global Outcomes (KDIGO) classification.

**Figure 2** shows the percentage of distribution of participants according to the number of risk factors and other complications among different risk categories based on KDIGO classification. Among all risk categories, participants with highest number of risk factors (n= >6) were significantly distributed among high/very high risk category. The percentage of distribution of number of risk factors and other complications (n= >6) in low risk category was 50% while moderately increased risk and high/very high risk categories showed 66.3% and 76.4% respectively (p=<0.001).

### Risk factors of DKD associated with the severity of kidney disease

**Figure 3:**
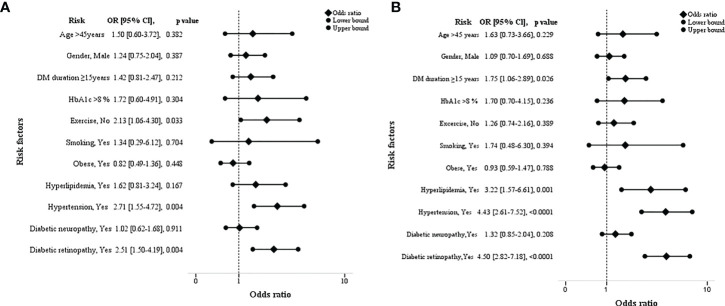
Forest plots showing the odds ratios of risk factors associated with the severity of kidney disease among type 2 diabetes. [low vs moderately increased risk **(A)** and low vs high/very high risk group **(B)**].

**Table 2** and **Figure 3** show the odds ratios of risk factors associated with the severity of kidney disease among T2D. In non-modifiable risk factors, DM duration ≥15 years was found to increase the odds of 1.75 in high/very high risk category (p=0.026). Furthermore, in high/very high risk category, presence of hyperlipidemia and hypertension showed an increase in odds of three times (OR=3.22, CI 95% 1.57-6.61); p=0.001) and four times (OR=4.43, CI 95% 2.61-7.52); p=<0.0001) respectively. In addition, presence of diabetic retinopathy remained significant with a higher OR of 4.5 (CI 95% 2.82-7.18); p=<0.0001) in high/very high risk category. In moderately increased risk category, lack of exercise (2.13, CI 95% 1.06-4.30); p=0.033), comorbidities; hypertension (OR=2.71, CI 95% 1.55-4.72); p=0.004), microvascular complication; presence of diabetic retinopathy (OR=2.51, CI 95% 1.50-4.19; p=0.004) had 2 times higher odds than low risk category. Diabetic retinopathy showed an increase in odds of 1.79 (p=0.02) in high/very high risk category than moderately increased risk group (**Supplementary Table 1**).

**Table 2:** Odds ratios of risk factors associated with the severity of kidney disease among patients with type 2 diabetes.

Parameters	Low risk (n=174)	Moderately increased risk (n=98)	OR (95% CI)	p value	Low risk (n=174)	High/very high risk (n=152)	OR (95% CI)	p value
	n (%)	n (%)			n (%)	n (%)		
**Non-modifiable risk factors**								
Age, >45 (years)	156 (89.7)	91 (92.9)	1.50 (0.60-3.72)	0.382	156 (89.7)	142 (93.4)	1.63 (0.73-3.66)	0.229
≤45 (years)	18 (10.3)	7 (7.1)	1.00		18 (10.3)	10 (6.6)	1.00	
Gender, Male	74 (42.5)	47 (48)	1.24 (0.75-2.04)	0.387	74 (42.5)	68 (44.7)	1.09 (0.70-1.69)	0.688
Female	100 (57.5)	51 (52)	1.00		100 (57.5)	84 (55.3)	1.00	
DM duration ≥15 (years)	117 (67.2)	73 (74.5)	1.42 (0.81-2.47)	0.212	117 (67.2)	119 (78.3)	1.75 (1.06-2.89)	0.026
<15 (years)	57 (32.8)	25 (25.5)	1.00		57 (32.8)	33 (21.7)	1.00	
**Modifiable risk factors**								
Obese, Yes	108 (62.4)	56 (57.7)	0.82 (0.49-1.36)	0.448	108 (62.4)	89 (61.0)	0.93 (0.59-1.47)	0.788
No	65 (37.6)	41 (42.3)	1.00		65 (37.6)	57 (39.0)	1.00	
HbA1c, >8 (%)	158 (91.3)	91 (94.8)	1.72 (0.60-4.91)	0.304	158 (91.3)	144 (94.7)	1.70 (0.70-4.15)	0.236
<8 (%)	15 (8.7)	5 (5.2)	1.00		15 (8.7)	8 (5.3)	1.00	
Exercise, No	134 (77.0)	86 (87.8)	2.13 (1.06-4.30)	0.033	134 (77.0)	123 (80.9)	1.26 (0.74-2.16)	0.389
Yes	40 (23.0)	12 (12.2)	1.00		40 (23.0)	29 (19.1)	1.00	
Smoking, Yes	4 (2.3)	3 (3.1)	1.34 (0.29-6.12)	0.704	4 (2.3)	6 (3.9)	1.74 (0.48-6.30)	0.394
No	170 (97.7)	95 (96.9)	1.00		170 (97.7)	146 (96.1)	1.00	
**Comorbidities**								
Hyperlipidemia, Yes	139 (79.9)	84 (86.6)	1.62 (0.81-3.24)	0.167	139 (79.9)	141 (92.8)	3.22 (1.57-6.61)	0.001
No	35 (20.1)	13 (13.4)	1.00		35 (20.1)	11 (7.2)	1.00	
Hypertension, Yes	95 (54.6)	75 (76.5)	2.71 (1.55-4.72)	0.004	95 (54.6)	128 (84.2)	4.43 (2.61-7.52)	<0.0001
No	79 (45.4)	23 (23.5)	1.00		79 (45.4)	24 (15.8)	1.00	
**Microvascular complications**								
Diabetic neuropathy, Yes	84 (48.3)	48 (49)	1.02 (0.62-1.68)	0.911	84 (48.3)	84 (55.3)	1.32 (0.85-2.04)	0.208
No	90 (51.7)	50 (51.0)	1.00		90 (51.7)	68 (44.7)	1.00	
Diabetic retinopathy, Yes	51 (29.3)	50 (51.0)	2.51 (1.50-4.19)	0.004	51 (29.3)	99 (65.1)	4.50 (2.82-7.18)	<0.0001
No	123 (70.7)	48 (49.0)	1.00		123 (70.7)	53 (34.9)	1.00	

Low risk vs moderately increased risk and low risk vs high/very high risk.

## Discussion

In this study, according to KDIGO classification, T2D participants with risk factors such as, hyperlipidemia, hypertension, DM duration ≥15 years and diabetic retinopathy showed a high/very high risk of CKD progression when compared with low risk category. While T2D participants with risk factors including, lack of exercise, hypertension, and diabetic retinopathy showed a moderately increased risk of CKD progression. In addition, participants with highest number of risk factors were significantly distributed among high/very high risk of CKD progression category.

T2D is characterized by persistent glycemia associated with insulin deficiency and insulin resistance. The pathogenesis of hyperglycemia is recognized in several organs such as liver, adipose tissue, intestine, kidney, central nervous system etc. (17, 18). The metabolic abnormalities in DKD includes glomerular hyper filtration, progressive albuminuria, declining glomerular filtration rate and eventually end stage renal disease. The metabolic changes associated with diabetes may alter kidney hemodynamics and promote glomerular hyperfiltration and albuminuria (19).

Progression of kidney disease is accelerated by a variety of modifiable and non-modifiable risk factors and associated comorbidities and unhealthy life styles such as, physical inactivity, uncontrolled blood pressure and blood glucose (20–22). The pathogenesis of the long-term persistent hyperglycemia has been associated with functional and structural changes in renal cells (23). A notable finding in this study is that among non-modifiable risk factors, only DM duration ≥15 years revealed a 1.75-fold increase in high/very high risk of CKD progression than low risk category. In a Saudi registry study, the prevalence of diabetic nephropathy was found to be increasing with duration of diabetes and the highest was reported in the duration >15 years (12). In a recent study, an adjusted hazard ratio showed that the patients with duration of diabetes <10 years had reduced the risk of diabetic nephropathy than those having longer duration of >10 years (24).

Hyperlipidemia has been implicated in the pathogenesis of CKD in diabetes. In this cohort high/very high risk category showed a three-fold increase in risk for hyperlipidemia than low risk category. A prospective cohort study of patients with T2D for a period of 5.8 years showed that elevated lipid levels were associated with increased development of albumin creatinine ratio (25). Furthermore, hypertension is widely known as important independent risk factor for CKD in diabetes (26, 27). This study reported a significant increased risk for hypertension among moderately increased category and high/very high risk category. In a previous study, hypertensive patients were found to be at a higher risk of developing DKD as compared to non-hypertensive subjects with an increase in odds of 1.67 (28).

It is well known that the prevalence of CKD and diabetic retinopathy increase proportionally to the duration of disease among T2D (29). This study reported a significant two-fold increased risk for diabetic retinopathy among moderately increased risk category whereas in high/very high risk category it was four-fold. This finding is in line with various population studies where it was reported that diabetic retinopathy is a known risk factors for diabetic nephropathy (12, 30, 31).

In general, physical activity would be effective in patients with diabetes which improves insulin sensitivity, endothelial dysfunction, cellular senescence and interstitial fibrosis which may cause end stage renal damage and renal dysfunction in diabetes (32–35). In this cohort, a significant two-fold increase in risk for lack of exercise was observed among moderately increased risk category than low risk. In an earlier study it was reported that CKD patients with T2D improved kidney function by 6-12% after twelve weeks exercise program (36).

In this study, the increase in number of risk factors (modifiable/non-modifiable) and other co-morbidities were linked with severity of CKD in T2D. In previous observations, risk factors such as family history of DKD, cigarette smoking, uncontrolled blood pressure, presence of low-grade inflammation, advanced glycation end products, lack of physical activity and hyperlipidemia had an influence on the progression to kidney disease in diabetes and combination of these risk factors have been identified as the ones that offer the greatest risk of development and progression of DKD (11, 37).

These study findings highlight the importance of using KDIGO classification to define CKD among patients with diabetes. Furthermore, in clinical practice, regular risk factor assessment could reduce the risk of kidney disease progression and cardiovascular disease. By implementing life style modifications, glycemic monitoring and pharmacological management may help to improve clinical outcomes of people with diabetes and CKD.

The limitations to this study include a cross-sectional-not longitudinal- analysis which impedes any contributing association between CKD and its risk factors. In addition, relatively small sample size and lack of up-to-date data on drugs made it difficult to determine its effect on clinical outcome.

## Conclusion

This study findings concludes that duration of diabetes ≥ 15 years, hyperlipidemia, hypertension and diabetic retinopathy have an increased prevalence of advanced CKD in T2DM. In addition to this, increased number risk factors could be an indicator for progression of CKD in T2D. Therefore, in high risk population, implementing screening of known risk factors at outset of disease may help to initiate treatment strategies at early phase and prevent or delay the progression of disease. Further studies in larger population are needed to determine the effect of these risk factors and complications in the progression of renal disease in T2D.

## Data availability statement

The raw data supporting the conclusions of this article will be made available by the authors, without undue reservation.

## Ethics statement

The studies involving human participants were reviewed and approved by Institutional Review Board (IRB) at College of Medicine, King Saud University (IRB/E-19/3969). Written informed consent for participation was not required for this study in accordance with the national legislation and the institutional requirements.

## Author contributions

All authors made substantial contributions to conception and design, acquisition of data, or analysis and interpretation of data; took part in drafting the article or revising it critically for important intellectual content; agreed to submit to the current journal; gave final approval of the version to be published; and agree to be accountable for all aspects of the work.
