# Supplementary material for: Risk factors of chronic kidney disease among type 2 diabetic patients with longer duration of diabetes
Source: Front Endocrinol (Lausanne). 2022 Dec 9;13:1079725. doi: 10.3389/fendo.2022.1079725 (PMC9780388; doi:10.3389/fendo.2022.1079725)
Supplement: Supplementary file 1 [file Table_1.docx]

| **Parameters** | **Moderately increased risk** | **High/ very high- risk** | **OR (95% CI)** | **p-value** |
| --- | --- | --- | --- | --- |
|  | (n=98) | (n=152) |  |  |
| **Non-modifiable risk factors** |  |  |  |  |
| Age, >45(years) | 91 (92.9) | 142 (93.4) | 1.09(0.40-2.9) | 0.86 |
| ≤45(years) | 7 (7.1) | 10 (6.6) | 1.00 |  |
| Gender, Male | 47 (48) | 68 (44.7) | 0.87(0.52-1.4) | 0.61 |
| Female | 51 (52) | 84 (55.3) | 1.00 |  |
| DM duration ≥15 (years) | 73 (74.5) | 119 (78.3) | 1.2(0.68-2.2) | 0.48 |
| <15 (years) | 25 (25.5) | 33 (21.7) | 1.00 |  |
| **Modifiable risk factors** |  |  |  |  |
| Obese, Yes | 56 (57.7) | 89 (61.0) | 1.14(0.67-1.9) | 0.61 |
| No | 41 (42.3) | 57 (39.0) | 1.00 |  |
| HbA1c, >8 (%) | 91 (94.8) | 144 (94.7) | 0.98(0.31-3.1) | 0.98 |
| <8 (%) | 5 (5.2) | 8 (5.3) | 1.00 |  |
| Exercise, No | 86 (87.8) | 123 (80.9) | 0.59(0.28-1.2) | 0.15 |
| Yes | 12 (12.2) | 29 (19.1) | 1.00 |  |
| Smoking, Yes | 3 (3.1) | 6 (3.9) | 1.30(0.31-5.3) | 0.71 |
| No | 95 (96.9) | 146 (96.1) | 1.00 |  |
| **Comorbidities** |  |  |  |  |
| Hyperlipidemia, Yes | 84 (86.6) | 141 (92.8) | 1.9(0.85-4.6) | 0.11 |
| No | 13 (13.4) | 11 (7.2) | 1.00 |  |
| Hypertension, Yes | 75 (76.5) | 128 (84.2) | 1.6(0.86-3.0) | 0.13 |
| No | 23 (23.5) | 24 (15.8) | 1.00 |  |
| **Microvascular complications** |  |  |  |  |
| Diabetic neuropathy, Yes | 48 (49) | 84 (55.3) | 1.2(0.77-2.1) | 0.33 |
| No | 50 (51.0) | 68 (44.7) | 1.00 |  |
| Diabetic retinopathy, Yes | 50 (51.0) | 99 (65.1) | 1.79(1.06-3.00) | 0.02 |
| No | 48 (49.0) | 53 (34.9) | 1.00 |  |

**Supp.table 1** Odds ratios of risk factors associated with the severity of kidney disease among patients with type 2 diabetes (moderately increased risk vs high/ very high-risk group**)**
